# Alox8 knockout exacerbates imiquimod-induced psoriasis-like inflammation

**DOI:** 10.1038/s41419-026-08727-9

**Published:** 2026-04-10

**Authors:** Megan A. Palmer, Rebecca Kirchhoff, Lisa Hahnefeld, Dominique Thomas, Mohammed A. F. Elewa, Xin You, Blerina Aliraj, Yvonne Benatzy, Andreas Weigert, Nils Helge Schebb, Bernhard Brüne

**Affiliations:** 1 Faculty of Medicine, Institute of Biochemistry I, Goethe University Frankfurt, Frankfurt, Germany; 2 Chair of Food Chemistry, School of Mathematics and Natural Sciences, University of Wuppertal, Wuppertal, Germany; 3 Goethe University Frankfurt, Faculty of Medicine, Institute of Clinical Pharmacology, Frankfurt, Germany; 4 Fraunhofer Institute for Translational Medicine and Pharmacology ITMP, Frankfurt am Main, Germany; 5 Fraunhofer Cluster of Excellence for Immune Mediated Diseases CIMD, Frankfurt am Main, Germany; 6 Department for Immunity of Inflammation, Mannheim Institute for Innate Immunoscience (MI3), Medical Faculty Mannheim, Heidelberg University, Mannheim, Germany; 7 German Cancer Consortium (DKTK), Partner Site Frankfurt, Germany

**Keywords:** Inflammation, Psoriasis

## Abstract

Lipoxygenases peroxidise polyunsaturated fatty acids, resulting in oxylipins, which may act pro- or anti-inflammatory. Arachidonate 15-lipoxygenase type B was shown to play a role in the resolution of keratinocyte inflammation and is upregulated in psoriasis. Its murine ortholog, arachidonate 8-lipoxygenase (Alox8), differs in regiospecificity in that it adds molecular oxygen to the 8th and not 15th carbon of arachidonic acid. This study aimed to determine if Alox8 plays a role in the resolution of murine imiquimod-induced psoriasis. Alox8 knockout (KO) mice, which are not commercially available, were generated with a functional KO targeting the enzyme’s active site. Untargeted Lipidomics revealed changes in the skin lipidome from both imiquimod-induced psoriasis as well as between wild-type and KO mice. Furthermore, LC-MS/MS revealed a functional KO with reductions in Alox8-specific oxylipins. Lipid peroxidation marker 4-hydroxynonenal was elevated in the epidermis of wild-type mice from imiquimod treatment, however, it was significantly reduced in Alox8 KO mice. Alox8 KO mice exhibited a thickened epidermis, resulting from reduced DNA damage and increased proliferation. Moreover, immune cell infiltration was enhanced in Alox8 KO mice, including a higher abundance of γδ T cells. Elevated cytokine levels of interleukin-17 and -22, accompanied by keratinocyte-produced C-X-C motif chemokine ligand 1, were detected in the skin of Alox8 KO mice. Additionally, cyclooxygenase 2 expression and prostaglandin E_2_ levels were enhanced in Alox8 KO mice. These data demonstrate an exacerbated and prolonged inflammatory psoriasis phenotype in Alox8 KO mice, implying that Alox8 aids in the resolution of murine psoriasis.

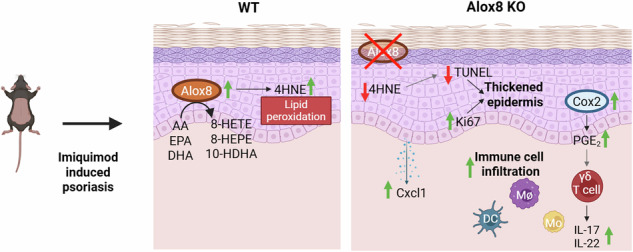

## Introduction

Lipoxygenases (LOX) are a group of enzymes that catalyse the insertion of molecular oxygen into polyunsaturated fatty acids. The resulting fatty acid hydroperoxides are promptly reduced to more stable hydroxy fatty acids, which can be both pro- and anti-inflammatory. In humans, LOX can be further categorised into 5-LOX (ALOX5), 12-LOX (ALOX12, ALOX12B), and 15-LOX (ALOX15, ALOX15B), named for which carbon atom of arachidonic acid (AA) is lipoxygenated [1]. Unlike its human ortholog, ALOX15B, Alox8 is unique in catalysing the lipoxygenation on the 8th carbon of AA [2]. Despite the differences in regiospecificity, similarities have been made to human disease [3]. Both enzymes have been associated with macrophage cholesterol homeostasis [4, 5], tumour suppressor functions [6], and involvement in lung inflammation [7, 8]. Nevertheless, ALOX15B is present in normal human prostate but is lacking in the mouse [9]. As there are currently no commercially available Alox8 knockout (KO) mice, we generated a functional KO targeting the active site of the enzyme.

Psoriasis is a chronic inflammatory skin disease characterised by thickened skin with excessive desquamation and erythema. It is pathologically driven by infiltration of immune cells, leading to interleukin (IL)-23/Th17 signalling axis, hyperproliferation, and abnormal differentiation of keratinocytes [10]. Moreover, lipid metabolism is dysregulated, provoking an abundance of pro-inflammatory ω-6 polyunsaturated fatty acid derivatives [11].

LOX-derived lipid mediators have been associated with skin inflammation. Whereas 12-LOX-derived 12-HETE and ALOX5-derived leukotrienes are pro-inflammatory chemoattractants [12], human 15-LOX-derived 15-HETE has anti-inflammatory effects and has been shown to inhibit ALOX12 activity [13]. Mice overexpressing Alox12b in the skin have increased IL17, leading to psoriasis-like skin inflammation [14]. Primary LOX products can undergo subsequent oxygenation to produce di- and trihydroxylated oxylipins such as resolvins, lipoxins, and protectins. Although the routes of enzymatic formation are not convincing and their occurrence in vivo is unclear [15], because they cannot be detected with state-of-the-art methods [16]. Nevertheless, intraperitoneal injection of resolvin D1 [17] and D5 in mice protected against UV-induced skin inflammation. Moreover, topical application of resolvins D1, D2, and D4 enhanced murine wound reepitheliasation [18]. Subcutaneous injection of protectin D1 [19], and topical application of lipoxin A4 [20] were shown to reduce inflammation in murine imiquimod (IMQ)-induced psoriasis models.

ALOX15B is upregulated in the epidermis [21] and sebaceous glands [22] of psoriasis patients. Moreover, knockdown of ALOX15B in human keratinocytes augmented inflammation [21]. In a murine IMQ psoriasis model, Alox8 expression was increased [23]. However, the role of Alox8 in the progression or resolution of psoriasis is unknown. This study aims to characterise the endogenous expression of Alox8 in different mouse tissues, along with determining if Alox8 plays a role in the resolution of murine psoriasis.

## Results

### *Alox8* RNA is expressed in the murine skin, ovary, and salivary gland

**Fig. 1:**
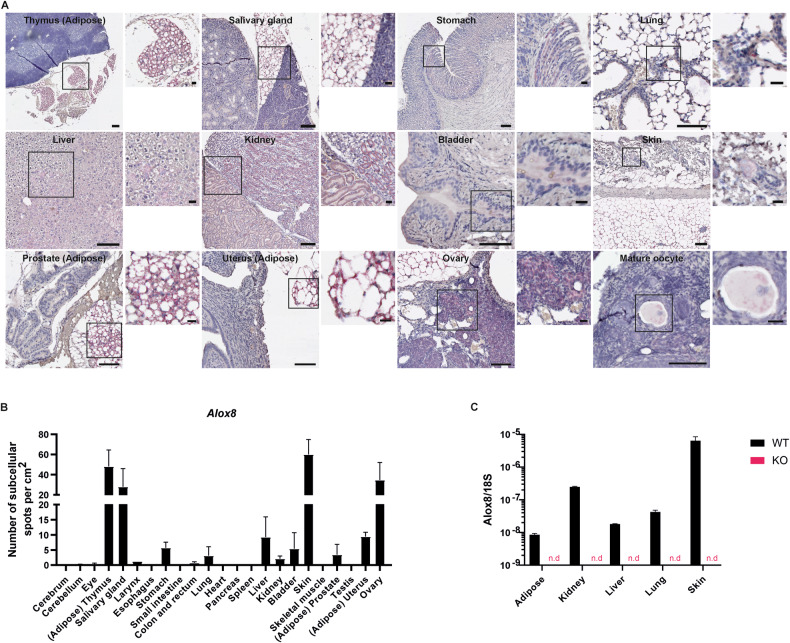
Alox8 expression in murine tissues. **A** Representative images of Alox8 RNA detected by BaseScope™ in situ hybridisation (fast red) in murine tissue array, nuclei counterstained with haematoxylin (blue). Scale bars 100 μm or zoomed regions 20 μm. **B** Quantification of subcellular spot detection per cm^2^ of tissue detected per tissue microarray core. **C** Gene expression analysis of *Alox8* in RNA extracted from bulk tissue of WT and Alox8 KO mice, relative to *18S*. Data are mean ± SEM, *N* = 2-3 for B and *N* = 3 for **C**.

To detect expression of Alox8 in murine tissue, BaseScope (in situ hybridisation) Assay was performed on a normal murine tissue microarray containing 22 tissues (Fig. 1A). Quantification of the number of RNA spots revealed higher levels of Alox8 expression in the cores containing thymus, salivary gland, skin and ovary (Fig. 1B). Further inspection of the thymus showed expression in the adipose tissue but not the thymus itself. Likewise, the lower levels of expression detected in the uterus and prostate were localised to adipose tissue. Within the salivary gland, *Alox8* expression was detected in the submandibular gland. Furthermore, *Alox8* expression was positive in the follicles and mature oocytes. Expression of *Alox8* in the skin was detected in the sebaceous glands and adipose tissue. Lower levels of *Alox8* were also detected in the stomach, lung, kidney, liver and bladder.

Next, we generated Alox8 KO mice and confirmed RNA expression from a selection of bulk tissue by qPCR (Fig. 1C). Like the in situ hybridisation results, the highest level of expression was detected in the skin of wild-type (WT) mice. Furthermore, expression was absent in the KO mice.

### KO of Alox8 in mice augments IMQ-induced psoriasiform dermatitis

**Fig. 2:**
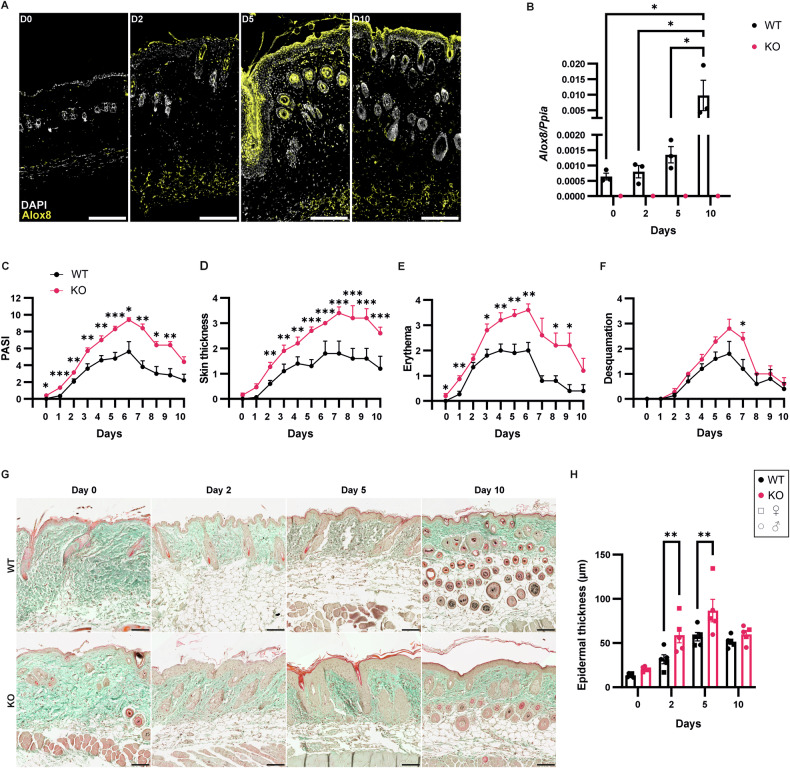
Alox8 KO elevates IMQ-induced psoriasis-like inflammation. IMQ was topically applied to the back skin of WT and Alox8 KO daily on day 0 for up to 6 times. Mice were sacrificed at day 0, 2, 5, or 10. **A** Immunofluorescence staining of Alox8 (yellow) in murine skin from WT, counterstained with DAPI (white), scale bar is 200 μm. **B** Gene expression analysis of *Alox8* relative to *Ppia* (*N* = 3). **C** Cumulative psoriasis area and severity index (PASI) score from **D** skin thickness, **E** erythema, and **F** desquamation (*N* = 20, 15, 10, or 5 for day 0, 1-2, 3-5, or 6-10, respectively). **G** Masson’s trichrome staining of murine skin from WT and KO mice, scale bar is 100 μm (*N* = 5). **H** Quantification of epidermal thickness from Masson’s trichrome images. Data are mean ± SEM, two-way ANOVA was performed; significance denoted by *p ≤ 0.05, **p < 0.01, ***p < 0.001.

Recently, we demonstrated that ALOX15B (the human ortholog of murine Alox8) is involved in the resolution of human keratinocyte inflammation, along with being upregulated in psoriasis [21]. We therefore verified whether Alox8 is also upregulated in murine psoriasis. Mice treated topically with IMQ showed a marked increase in Alox8 expression in the stratum corneum, stratum granulosum, adipocytes, and inner root sheath of the hair follicle (Fig. 2A). Furthermore, an increase in *Alox8* RNA expression was confirmed in the skin of WT mice, including significant increases at day 10 (Fig. 2B).

Given our previous results showing RNAi silencing of ALOX15B in human keratinocytes increased inflammation [21], we hypothesised that Alox8 KO mice would also have a more inflammatory phenotype. Elevated psoriasis area and severity index (PASI) scoring in Alox8 KO mice was apparent in comparison to WT mice (Fig. 2C). Significant increases in skin thickness and erythema were observed after day 2 and 3, respectively (Fig. 2D+E), whereas desquamation was only increased in KO mice at day 7 (Fig. 2F). Furthermore, resolution of inflammation remained incomplete at day 10 in KO mice, with elevated erythema and thickened skin. Next, Masson’s trichrome staining was performed to analyse tissue morphology (Fig. 2G). No changes in collagen production (green) were detected. However, significant increases in epidermal thickness were observed by image analysis at days 2 and 5 for both male (circles) and female (squares) mice (Fig. 2H).

### Alox8 KO alters the skin lipidome

**Fig. 3:**
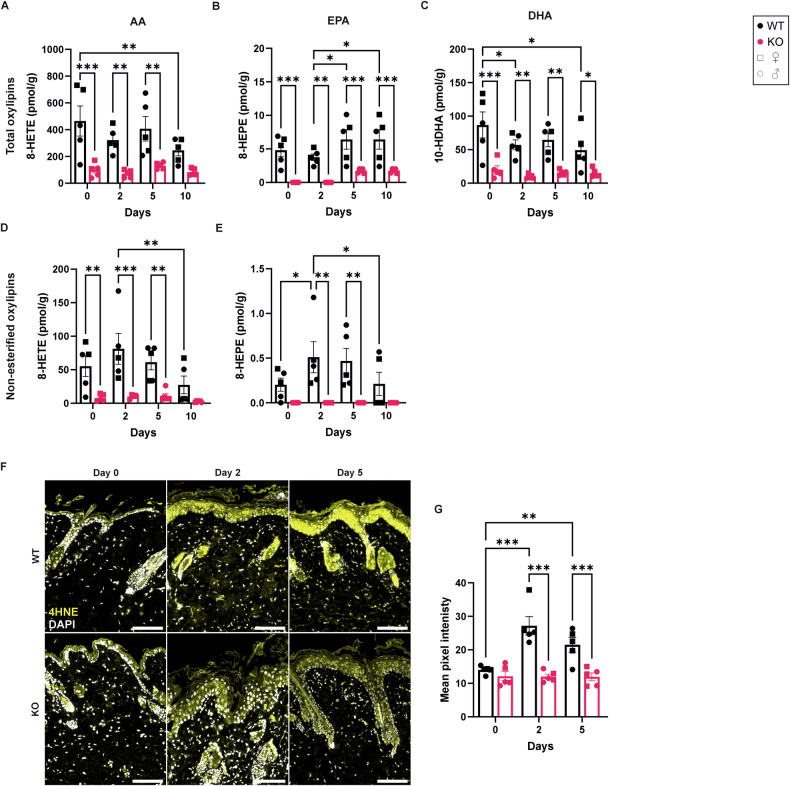
Alox8 KO mice reduce Alox8-specific oxylipins in mouse skin. IMQ was topically applied to the back skin of WT and Alox8 KO daily on day 0 for up to 6 times. Mice were sacrificed at day 0, 2, 5, or 10. Analysis of 8-LOX (**A**–**C**; total oxylipins, **D**, **E**; non-esterified). **F** Immunofluorescence staining of lipid peroxidation marker 4HNE (yellow) and DAPI (white), scale bars are 100 μm. **G** Image analysis of mean pixel intensity for the epidermis area. Data are mean ± SEM, *N* = 5, two-way ANOVA was performed; significance denoted by *p ≤ 0.05, **p < 0.01, ***I < 0.001.

To confirm a functional KO of Alox8, analysis of oxylipins was performed. Alox8-specific oxylipins [24] derived from AA: 8-hydroxyeicosatetraenoic acid (8-HETE), eicosapentaenoic acid: 8-hydroxyeicosapentaenoic acid (8-HEPE), and docosahexaenoic acid (DHA): 10-hydroxydocosahexaenoic acid (10-HDHA) were significantly reduced at day 0, 2, 5, and 10 (Fig. 3A–C). Likewise, analysis of Alox8-specific non-esterified oxylipins showed that 8-HETE (Fig. 3D) was significantly reduced and 8-HEPE (Fig. 3E) was below the lower limit of detection in Alox8 KO mice. Non-esterified (Fig. 3D+E) but not total (esterified and non-esterified) oxylipins (Fig. 3A+B) had a trend increase in 8-HETE and a significant increase in 8-HEPE between day 0 and day 2 in WT mice. Furthermore, Kakularam et al. [24] demonstrated in vitro that murine Alox8 purified from *E. Coli* could produce small quantities of 15-HETE, 15-HEPE, 17-HDHA, and 7-HDHA. No changes in 15-LOX products were detected (Fig. S1); however, 7-HDHA showed a significant reduction comparing WT and KO mice at day 2 but not between day 0 and day 2 in KO mice (Fig. S1).

Moreover, there were no changes in protein abundance of Alox5 (Fig. S2A) and Alox15 (Fig. S2B), indicating that the trend reduction in 7-HDHA may be due to the loss of Alox8. No changes were detected for other oxylipins comparing WT and KO mice (Fig. S1).

Given the increase in Alox8 RNA and protein expression in murine psoriasis, an increase in Alox8-specific oxylipins was expected. As there were no significant increases detected by LC/MS-MS, immunohistochemistry of the lipid peroxidation marker 4-hydroxynonenal (4HNE) was performed. 4HNE staining was predominantly localised to the epidermis and sebaceous glands (Fig. 3F), aligning with Alox8 expression pattern. Analysis of the epidermal 4HNE staining showed significant increases at days 2 and 5 compared to day 0 in WT mice (Fig. 3G). Moreover, these increases were not detected in KO mice, suggesting that Alox8 may be responsible for the increase in lipid peroxidation in the epidermis upon IMQ treatment.

**Fig. 4:**
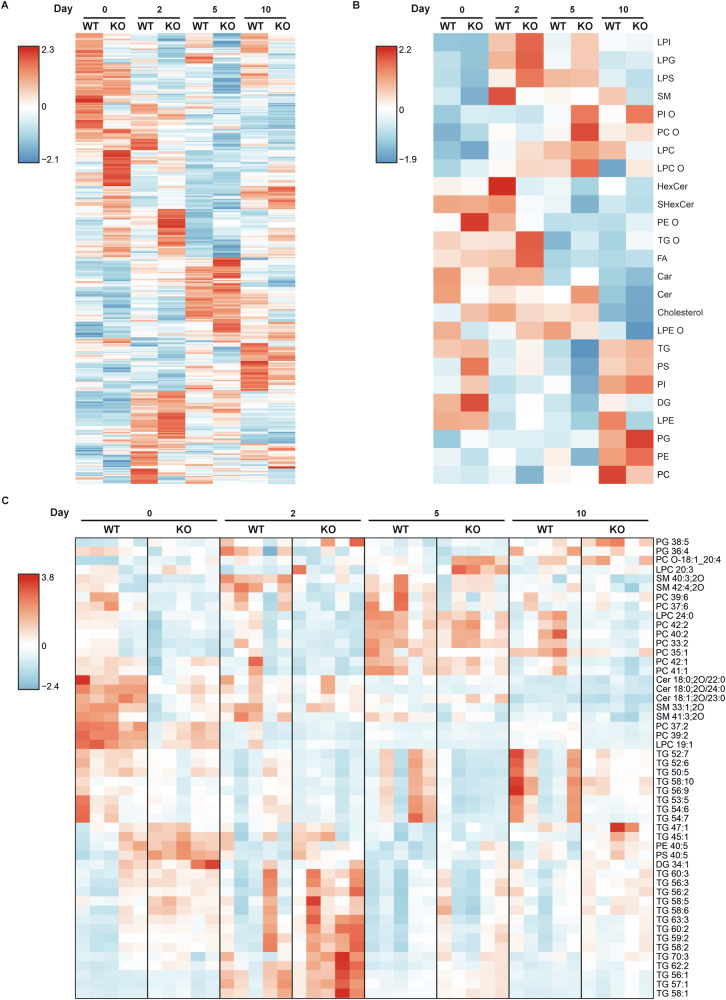
Alterations in the lipidome between WT and Alox8 KO skin and IMQ treatment. IMQ was topically applied to the back skin of WT and Alox8 KO daily on day 0 for up to 6 times. Mice were sacrificed at day 0, 2, 5, or 10. Untargeted lipidomic analysis represented by heatmaps for **A** mean abundance of lipid per treatment group from all lipid species detected or **B** summed lipids by lipid class. **C** Top 50 significantly altered lipids detected by two-way ANOVA, each rectangle represents one individual biological replicate (*N* = 5).

Lipids are essential in cutaneous biology, with particular importance to the permeability barrier, along with functioning as signalling molecules that modulate keratinocyte differentiation and inflammatory processes [25]. Therefore, untargeted lipidomics was performed from skin of WT and KO mice to detect changes in lipid species. Both Alox8 KO and IMQ-induced psoriasis altered the lipidome in murine skin (Fig. 4A). Lipids were then grouped to see changes in abundance (Fig. 4B). Lysophosphatidylinositol, lipophosphoglycan, lipopolysaccharide, lysophosphatidylcholine, and sphingomyelins were upregulated through IMQ treatment. Ceramides, hexocosyleramides, acylcarnitines, along with ether-linked phosphatidylethanolamine and triglycerides were reduced in Alox8 KO skin at days 5 and 10. Several phospholipid classes and triglycerides showed an increase in abundance in the skin of day 10 mice from both genotypes.

Figure 4C shows the top 50 significantly regulated lipids. A selection of phosphatidylcholines was upregulated after 5 days of IMQ treatment. Whereas phosphatidylcholine 37:2 and 39:2 show downregulation with IMQ treatment in both genotypes of mice. Ceramides, 18:0;2O/22:0, 18:0;2O/24:0, and 18:1;2O/23:0, were significantly reduced in KO mice compared to WT, along with additional reductions from IMQ treatment. Some triglycerides are significantly upregulated in KO mice compared to WT mice, while others are downregulated. Moreover, the abundance of polyunsaturated fatty acids linoleic acid, AA, and DHA (Fig. S3A–C) was individually examined from the untargeted lipidomics results. A significant increase in AA (Fig. S3B) was detected on day 2 in KO mice in comparison to both day 0 and WT mice. These changes highlight disruption to the skin lipidome in KO mice beyond the Alox8-specific enzymatic activity.

### Alox8 KO upregulates Cox2 and PGE_2_ production

**Fig. 5:**
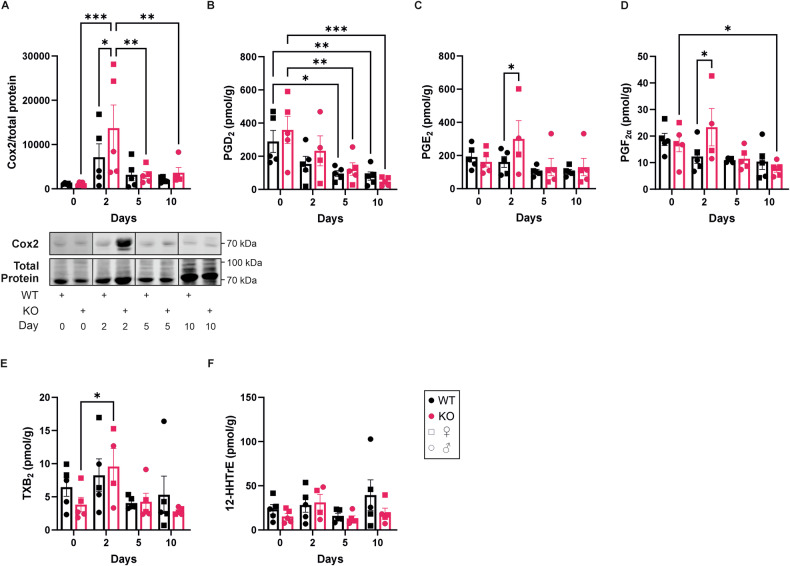
Cox2 expression and prostaglandin synthesis are upregulated in KO mice after IMQ treatment. IMQ was topically applied to the back skin of WT and Alox8 KO daily on day 0 for up to 6 times. Mice were sacrificed at day 0, 2, 5, or 10. **A** Western analysis of Cox2 relative to total protein stain (*N* = 5). Analysis of Cox specific oxylipins **B** PGD2, **C** PGE2, **D** PGF2α, **E** TBX2, and **F** 12-HHTrE (*N* = 5, except for KO day 2, *N* = 4). Data are mean ± SEM, two-way ANOVA was performed; significance denoted by *P ≤ 0.05, **P < 0.01, ***P < 0.001.

Cyclooxygenase 2 (Cox2) expression is upregulated in the skin under inflammatory conditions [26], provoking the production of prostaglandin (PG) E_2_ [27]. Therefore, Cox2 protein levels were determined by Western analysis (Fig. 5A). Cox2 was significantly increased at day 2 in comparison to day 0 in Alox8 KO mice and significantly higher than in WT mice at day 2. LC/MS-MS analysis of PGs revealed no change in anti-inflammatory PGD_2_ (Fig. 5B), but significant increases in PGE_2_ and PGF_2α_ between WT and KO mice at day 2 (Fig. 5C+D). Other Cox2 products, thromboxane B_2_ (Fig. 5E) and 12-hydroxyheptadecatrienoic acid (Fig. 5F), remained unchanged between WT and KO mice.

### Alox8 KO increases γδ T cell infiltration and IL-17 production

**Fig. 6:**
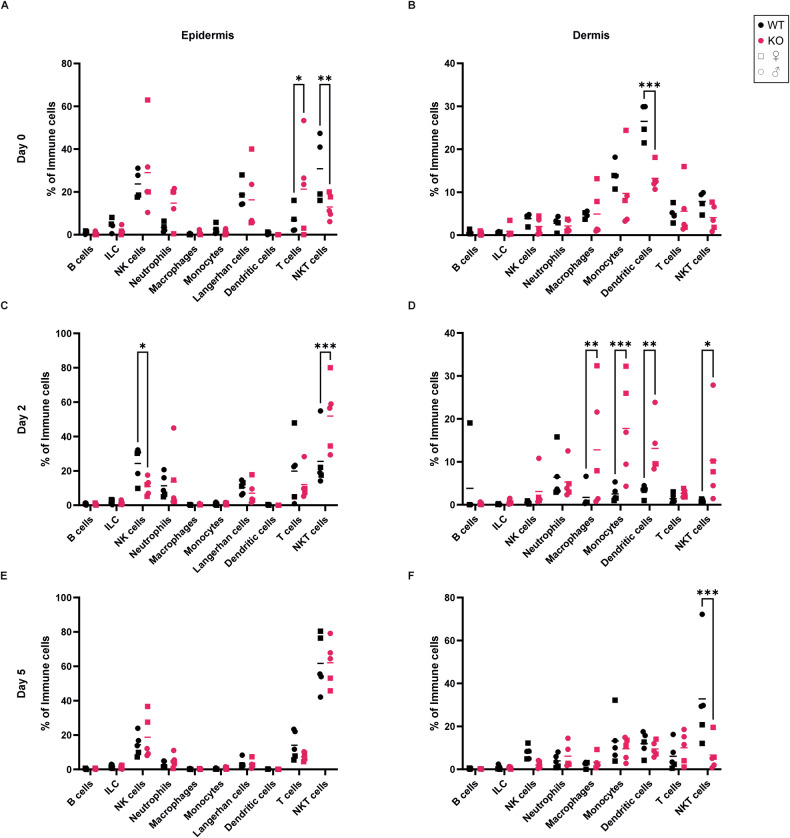
Immune cell profile in epidermis and dermis of murine skin. IMQ was topically applied to the back skin of WT and Alox8 KO daily on day 0 for up to 5 times. Mice were sacrificed at day 0, 2, or 5. Flow cytometry analysis of single cell suspensions of epidermis (**A**, **C**, **E**) or dermis **(B**, **D**, **F**). Percentages of immune cells relative to CD45+ cells. Data are mean ± SEM, *N* = 5 (except for WT day 0; *N* = 4), two-way ANOVA was performed; significance denoted by *P ≤ 0.05, **P < 0.01, ***P < 0.001.

**Fig. 7:**
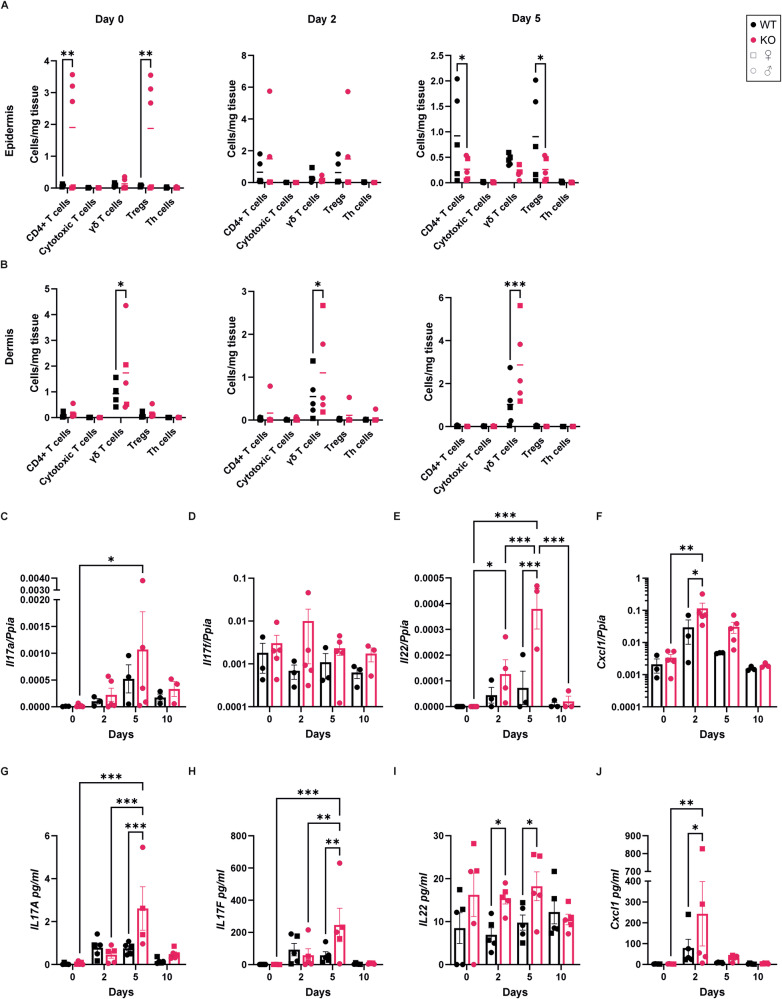
γδ T cells are elevated in the dermis of Alox8 KO mice with psoriasis-like inflammation. IMQ was topically applied to the back skin of WT and Alox8 KO daily on day 0 for up to 5 times. Mice were sacrificed at day 0, 2, 5, or 10. Flow cytometry analysis T cell subtypes of single cell suspensions of **A** epidermis or **B** dermis. Percentages of immune cells relative to CD45+ cells. **C**–**F** Gene expression and (**G**–**J**) cytokine analysis via cytometric bead array of (**C** + **G**) IL17a, (**D** + **H**) IL17f, (**E** + **I**) IL22, and (**F** + **J**) Cxcl1. Data are mean ± SEM, *N* = 3–5, two-way ANOVA was performed; significance denoted by *P ≤ 0.05, **P < 0.01, ***P < 0.001.

Psoriasis is characterised by infiltrating immune cells; therefore, single-cell suspensions from the epidermis and dermis were generated for flow cytometry analysis (Figs. 6, S4, and S5). In untreated mice (day 0), a significant increase in T cells and decrease in natural killer T cells was observed in the epidermis (Fig. 6A), with reductions in dendritic cells in the dermis of KO mice (Fig. 6B). With the onset of psoriasis (day 2), significant increases in natural killer T cells in the epidermis (Fig. 6C), and macrophages, monocytes, dendritic cells and natural killer T cells were found in the dermis of KO mice (Fig. 6D). No changes were detected between immune cells in the epidermis (Fig. 6E), however there was a significant reduction in the number of NKT cells in the dermis on day 5 (Fig. 6F). Further analysis into subsets of T cells showed an increase in regulatory T cells in the epidermis at day 0, this increase was only detected from male mice. Furthermore, there was a significant reduction in regulatory T cells in the epidermis (Fig. 7A) at the peak of inflammation (day 5) and an increase in γδ T cells in the dermis (Fig. 7B) at all time points.

Next, gene expression of key cytokines in the progression of psoriasis was analysed. Increases in T cell-produced cytokines *Il17a, Il17f* and *Il22* were found, with significant increases in *Il17a* in Alox8 KO mice between day 0 and 5 (Fig. 7C), but no significant changes in *Il17f* (Fig. 7D). *Il22* was significantly increased at both day 2 and 5 in Alox8 KO mice in comparison to day 0, and between WT and KO at day 5 (Fig. 7E). Additionally, keratinocyte-produced C-X-C motif chemokine ligand 1 (*Cxcl1)* was significantly increased between days 0 and 2 for KO mice and at day 2 between WT and KO mice (Fig. 7F). Next, cytometric bead arrays were used to confirm these changes at the protein level. IL-17A (Fig. 7G) and IL-17F (Fig. 7H) were significantly increased at day 5 in KO mice compared to WT and KO day 0 mice. Similarly, IL-22 was significantly increased at both days 2 and 5 in KO mice compared to WT (Fig. 7I). C-X-C motif chemokine ligand 1 protein was significantly enhanced in day 2 KO mice compared to both day 0 and WT mice (Fig. 7J).

### TUNEL positive cells are reduced, and proliferation is increased in the skin of Alox8 KO mice

**Fig. 8:**
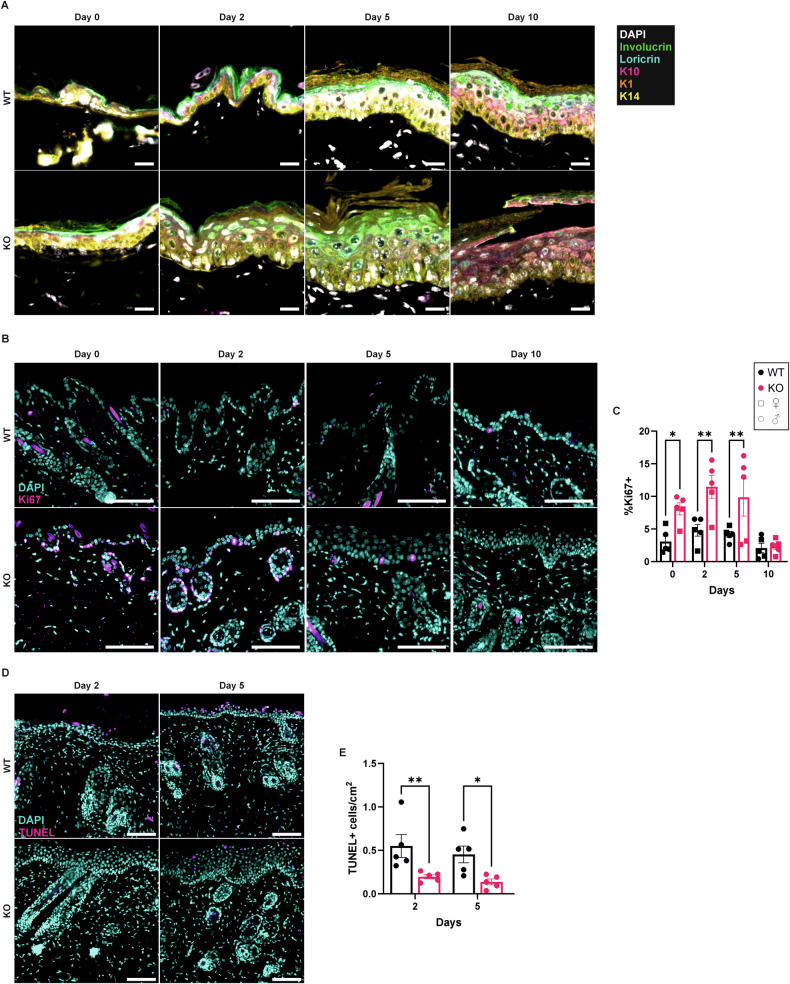
Differentiation and proliferation markers in WT and Alox8 KO skin. IMQ was topically applied to the back skin of WT and Alox8 KO daily on day 0 for up to 6 times. Mice were sacrificed at day 0, 2, 5, or 10. **A** Immunofluorescence images of differentiation markers in the epidermis, scale bar 20 μm. **B** Immunofluorescence images of proliferation marker ki67 and **C** image analysis of percentage Ki67+ cells in the epidermis. **D** Immunofluorescence images of TUNEL and **E** image analysis of the number of TUNEL+ cells per cm^2^ in the epidermis. Images from B + C are counterstained with DAPI (cyan), scale bars 100 μm. Data are mean ± SEM, *N* = 5, two-way ANOVA was performed; significance denoted by *P ≤ 0.05, **P < 0.01.

In addition to immune cell infiltration, abnormal keratinocyte differentiation occurs in psoriasis. Multiplexed fluorescence immunohistochemistry staining of differentiation markers keratin 14 (K14), K1, K10, involucrin, and loricrin was performed (Fig. 8A+S6). Image analysis showed a significant increase in stratum basal marker K14 in untreated KO mice; however, no change from IMQ treatment (Fig. S6B). K14 expression in KO mice was shown throughout the epidermis, whereas in WT mice, the localisation was predominantly in the stratum basale. Stratum spinosum markers K1 and K10 showed similar trends in intensity, with K1 significantly increased on day 0 KO mice (Fig. S6C) and K10 significantly decreased on day 2 (Fig. S6D). Involucrin expression in normal skin is associated with the stratum spinosum and granulosum layers, and no alterations in intensity (Fig. S6E) or expression pattern were noted between WT and KO mice. Loricrin expression is typically localised to the stratum corneum. While increases in loricrin intensity were detected only in KO day 0 mice compared to WT (Fig. S6F), abnormal expression patterns can be detected in KO mice (Fig. S6A). Collectively, these data indicate that Alox8 KO reduces keratinocyte differentiation.

Hyperproliferation of the epidermis is a typical characteristic of psoriasis. Therefore, marker of proliferation Ki67 was detected by immunofluorescence staining (Fig. 8B). Image analysis for the percentage of Ki67+ nuclei revealed significant increases at days 0, 2, and 5 between KO and WT mice (Fig. 8C). Next, mouse skin was probed for terminal deoxynucleotidyl transferase dUTP nick end labeling (TUNEL) (Fig. 8D). Alox8 KO mice had significantly fewer TUNEL-positive nuclei at both day 2 and 5 (Fig. 8E).

To determine whether the shaving procedure on day -1 altered the integrity of the skin, immunofluorescence staining of K14 was performed. K14 was localised to basal keratinocytes in both WT and KO mice in unshaved mice (Fig. S7A). The presence of suprabasal K14 in Alox8 KO mice at day 0 (Fig. 8A+S6) indicates a hyperproliferative state of Alox8 mice after shaving. Given the changes in the lipid composition of the skin, investigations into the skin barrier were performed to determine if IMQ could penetrate deeper in Alox8 KO mice. Lucifer yellow was unable to permeate the stratum corneum in both WT and KO mice (Fig. S7B). Moreover, adherens junction marker E-cadherin showed no difference in expression pattern between WT and Alox8 KO mice (Fig. S7A). These data indicate that the increased inflammatory phenotype in Alox8 KO mice is not due to greater IMQ penetration from an impaired barrier function. Nevertheless, suprabasal K14 expression in Alox8 mice may indicate that the 24-hour period between shaving and the first IMQ application was insufficient to reduce inflammation from mechanical irritation to baseline.

## Discussion

Expression of Alox8 was first described in murine skin upon phorbol ester treatment [2, 28]. Here we demonstrate that IMQ-induced psoriasis upregulated Alox8 at both protein and RNA levels in WT mice. These results confirm Wang et al. [23] previous findings where Alox8 was upregulated from 6 days of topical IMQ treatment of mice’s ears. Likewise, Alox8 is upregulated in the spontaneous dermatitis present in NFκB inhibitor alpha (IκB-α) deficient mice [29]. Furthermore, we recently demonstrated a role for ALOX15B in the resolution of psoriasis [21]. Collectively, these data led to the hypothesis that Alox8 aids in the resolution of psoriasis. Indeed, Alox8 KO mice exhibited a similar phenotype, with enhanced inflammation after IMQ-induced psoriasis, characterised by increased skin thickness, T cell infiltration, and IL-17 production.

Analysis of oxylipins in the skin of Alox8 KO mice revealed Alox8-specific reductions in 8-HETE, 8-HEPE and 10-HDHA. Additionally, a trend in reductions was seen for 7- and 17-HDHA, aligning with in vitro enzymatic studies showing Alox8 produced small amounts of these oxylipins from DHA [24]. However, due to biological variation, further experiments with more mice are needed to conclude Alox8 in vivo activity for alternative DHA products. 15-HETE and 15-HEPE also showed a trend but were not significantly reduced. In fact, Alox15 protein was reduced in the skin of IMQ-treated BALB/c mice [20]. Yet, these results could not be reproduced in our C57BL/6N mice. Alox15 produces a higher ratio of 12-LOX products in mice than in humans, and no changes in 12-LOX products were found. Moreover, Alox8 produces small amounts of 15-LOX products [24], particularly when complex lipids are given as substrate [30]. Therefore, the slight reductions in 15-LOX products may also be from the loss of Alox8 in these KO mice. Although there was a significant increase in Alox8 expression, only a small increase in free 8-HEPE was detected on day 2 in WT mice. However, lipid peroxidation marker 4HNE was significantly increased in the epidermal keratinocytes. Given that 4HNE was reduced in the Alox8 KO epidermis, the increased Alox8 expression detected with IMQ treatment in WT mice may contribute to localised changes in oxylipins and lipid peroxidation.

Elevated 4HNE has previously been detected in the keratinocytes [31] and the lesional skin of human psoriatic patients [32]. Ferroptosis inhibitor ferrostatin-1 was shown to alleviate IMQ-induced psoriasis with reduced 4HNE levels [32]. Therefore, impaired lipid peroxidation could be interpreted as a less inflammatory phenotype, which was not seen in Alox8 KO mice. Additionally, 4HNE treatment in Chinese hamster ovary cells has been shown to induce apoptosis through cleavage of caspase-3 and poly ADP-ribose polymerase 1 [33, 34], along with the induction of DNA damage marker γH2AX in dermal fibroblasts [35]. Since psoriasis is associated with downregulation of apoptosis, reduced 4HNE levels found in Alox8 KO mice may be associated with reduced TUNEL positive cells detected at both day 2 and 5, and therefore, explain the thickened skin observed in these mice. Additionally, HNE is associated with inhibited AKT signalling [36], which is important for regulating proliferation and differentiation in the skin.

Alox8 KO increased both Cox2 expression and PGE_2_ levels in the mouse skin. PGE_2_ was shown to enhance IL-17 production in γδ T cells in vitro. Furthermore, intraperitoneal PGE_2_ elevated IL-17 levels and γδ T cells in murine IMQ-induced psoriasis model, along with skin thickening [37]. Moreover, IL-17 and γδ T cells were both elevated in Alox8 KO mice. Both PGE_2_ and its receptors (EP2/EP4) signalling have been linked with Th17 cell infiltration. Moreover, EP2 KO or EP4 antagonist AS1954813 suppresses IMQ-induced psoriasis. Likewise, Cox inhibitors suppress inflammation in both an IL-23-induced murine psoriasis model [38] and IMQ-induced psoriasis [39]. Interestingly, reduction of Alox8 in lymphoma cells via chromosome 17p deletions or shRNA also showed an increase in PGs and *Ptgs2* (Cox2 gene) [40]. Our data support the idea proposed by Qi et al. [40] that loss of Alox8 can lead to an imbalance of PGE_2_ synthesis. However, this change was only detected at the onset of inflammation.

Lipidomic analysis showed a disruption in the skin lipidome, both from IMQ-induced psoriasis and loss of Alox8. Sphingolipids make up almost half of the lipids in the epidermis and are an important component of the epidermal permeability barrier [41]. Alox8 KO mice had reduced sphingomyelins and ceramides in comparison to WT skin. Moreover, sphingomyelin and ceramide levels were increased and decreased, respectively, in IMQ-treated WT skin, which aligns with reports in human psoriasis [42–44]. Ceramides have been associated with inhibiting proliferation [45] along with inducing differentiation [46] and apoptosis [47, 48]. In contrast, sebum is largely comprised of triglycerides [49], and the increased levels of triglycerides detected in Alox8 KO mice may reflect the localisation of Alox8 in the sebaceous gland detected with in situ hybridisation. Collectively, the lipid changes detected in the skin of Alox8 KO mice imply a function in the regulation of normal keratinocyte biology.

Peroxisome proliferator-activated receptors (PPARs) are activated by oxylipins and fatty acids. Moreover, Alox8-specific oxylipins 8-HETE and 8-HEPE have been shown to activate all PPAR isoforms [50, 51]. PPARα is important for epidermal barrier formation in utero, along with inhibiting proliferation and inducing differentiation in the murine epidermis [52, 53]. Both PPARα activation via 8-HETE in keratinocytes, along with transgenic mice overexpressing Alox8 in the skin, enhanced expression of the differentiation marker K1 [54]. Likewise, PPARγ agonists induce expression of keratinocyte differentiation markers and epidermal barrier recovery in mice [55]. However, PPARβ/δ is increased in psoriasis, whereas PPARα and PPARγ are not. Activation of the PPARβ/δ pathway promotes differentiation and inhibits proliferation [56], and PPARβ^+/-^ mice have increased Ki67 in the epidermis [57]. Aligning with our results that Alox8 KO mice have increased Ki67+ cells in the epidermis, inducible expression of both Alox8 and ALOX15B in murine keratinocytes inhibits proliferation [58]. Likewise, incubation of keratinocytes with 8(S)-HETE or 15(S)-HETE reduced the number of bromodeoxyuridine-positive cells [58]. Collectively, these data suggest Alox8 and its products may play a role in the regulation of keratinocyte growth and differentiation through PPAR signalling.

In conclusion, loss of Alox8 exacerbates skin thickening in IMQ-induced psoriasiform dermatitis mediated via increased proliferation and decreased TUNEL positivity. Additionally, immune cell infiltration was higher in Alox8 KO mice with increased monocytes, macrophages, dendritic cells and γδ T cells. This increase correlates with an increase in pro-inflammatory cytokines IL-17A, IL-17F, and IL-22, along with keratinocyte-produced chemokine Cxcl1. Contrarily, in WT mice significant changes were not apparent for IL-17A and IL-22 protein levels upon IMQ treatment, this may be due to the C57BL/6 strain that was shown to have a lack of consistent increase in IL-17A and IL-22 in comparison to BALB/c mice [59]. In Alox8 KO mice, an additional increase in pro-inflammatory Cox2 and PGE_2_ was detected at day 2. These data indicate a role for Alox8 and its products in the resolution of murine psoriasis. Furthermore, we showed for the first time expression of Alox8 in the ovary, uterus, and sublingual gland; therefore, further investigation on the role of Alox8 in these tissues is warranted.

## Materials and methods

### Animal experiments

Alox8 KO mouse strain was generated by GenOway (Lyon, France) using C57BL/6N mice. A targeting vector with loxP sites flanking exons 6–8 (Fig. S8A) was electroporated into embryonic stem cells. Embryonic stem cells were then injected into blastocysts for chimera generation. Heterozygous mice expressing the recombined Alox8 locus were bred with C57BL/6N Cre-deleater mice to generate the parent strain for breeding knock-out mice. WT mice of the same strain (C57BL/6N) were purchased from Charles River Laboratories (Wilmington, Massachusetts, USA). Genotyping was confirmed from ear markings via PCR as indicated in Fig. S8A. Animal experiments were performed in accordance with the Hessian animal welfare and use committee under FU/2053.

All mice were aged between P56 and P65 at the beginning of experiments to ensure that the hair cycle was in late telogen [60]. The back skin of mice was shaved on day -1 using an electric razor (Isis, Aesculap Schermaschinen, Suhl, Germany). 62.5 mg of cream containing 5% IMQ (Aldara, Viatris Healthcare, Bad Homburg, Germany) was applied topically to mouse skin for 2, 5, or 6 consecutive days. PASI scoring was performed daily to analyse erythema (redness), skin thickness, and desquamation (scaling) up to day 10. Representative images of scoring are provided in Fig. S8B. Skin thickness was measured using callipers, scores for skin thickness were as follows 0: <0.3 mm, 1: 0.3–0.49 mm, 2: 0.5–0.69 mm, 3: 0.7–0.89 mm, 4 > 0.9 mm. Mice were subsequently sacrificed at day 0 (untreated control), 2, 5, or 10 for further analyses (Fig. S8C). 5 mice were used per experimental group, with 2–3 females/males per group. Group sizes were chosen based on prior experience with the model. Mice were randomly assigned to experimental groups, and samples for further analysis were blinded upon collection. No mice were excluded from the study.

### Gene expression

RNA was isolated from tissue samples using ReliaPrep™ RNA miniprep system (Promega, Madison, Wisconsin, USA) as per manufacturer’s instructions. Tissue was lysed in 500 μl of buffer LBA containing 1-Thioglycerol and homogenised using Gentle MACS M tube with a gentleMACS Octo Dissociator (Miltenyi Biotec, Bergisch Gladbach, Germany) with programme RNA_01. Maxima First Strand cDNA Synthesis Kit (ThermoFisher Scientific, Waltham, Massachusetts, USA) was used to convert RNA into cDNA. 10 ng cDNA was used for qPCR using Power up SYBR green master mix (Applied Biosystems™, ThermoFisher). Analysis was performed using the ΔΔCT method using *Ppia* as a reference gene. Primer (Biomers.net, Ulm, Germany) sequences are available in supplementary information.

### Western blotting

Tissue samples were lysed in RIPA buffer (50 mM Tris-HCl, pH 7.4, 150 mM NaCl, 1 mM EDTA, 10 mM NaF, 0.1% sodium dodecyl sulfate, 0.5% sodium deoxycholate, 1% Triton X, 1 mM phenylmethylsulfonyl fluoride, and protease inhibitors). Samples were homogenised using Gentle MACS M tube with gentleMACS Octo Dissociator with programme Protein_01. 50 μg of protein was run on 10% tri-glycine gels and transferred onto nitrocellulose membranes using a Trans-Blot Turbo transfer system (Bio-Rad, Hercules, California, USA). Revert™ 700 Total Protein Stain (LI-COR, Lincoln, Nebraska, USA) was used as per manufacturer recommendations. Blocking was performed with 5% bovine serum albumin in TBST for 1 h before incubations with primary antibodies (supplementary information) at 4 °C overnight. Membranes were incubated with either IRDye® 800CW or 680RD Goat anti-Rabbit IgG secondary antibodies for 1 h at room temperature. Odyssey CLx (LI-COR) was used to scan membranes, and densitometry analysis was performed in ImageStudio ™ Lite (LI-COR).

### Oxylipin analysis

Tissue samples were washed once with PBS and dissected into 10-20 mg pieces, then snap frozen in liquid nitrogen. Tissues were homogenised with a Bead Ruptor Elite (Biolabproducts, Bebensee, Germany) using 5 mm stainless steel balls 2–6 times for 2 min at 25 Hz in ice-cold isopropanol (total oxylipins) or methanol (non-esterified oxylipins) containing antioxidants and internal standards. Proteins were precipitated with ice-cold isopropanol for 30 min at −80 °C, followed by centrifugation at 20,000 × *g* for 10 min at 4 °C. For total oxylipins, supernatants were saponified with 0.6 M potassium hydroxide in methanol/H_2_O (75/25 v/v) for 30 min at 60 °C, followed by neutralisation [61]. Following purification using solid phase extraction, total or non-esterified oxylipins were quantified by LC-MS/MS as described [62–65].

### Lipidomics

Tissue samples were washed once with PBS and dissected into 10-20 mg pieces, then snap frozen. Prior methyl-tert-butyl-ether based lipid extraction, tissue samples were homogenized in 25% ethanol containing 10 µM indomethacin using a Precellys 24-Dual tissue homogenizer (Bertin Technologies, Montigny-le-Bretonnex, France) maintained at <6 °C. 40 µL of the resulting homogenate was extracted as previously described [66, 67]. Data were acquired on a Vanquish Horizon UHPLC system coupled to an Orbitrap Exploris 480 (both Thermo Fisher Scientific, Dreieich, Germany) with a Zorbax RRHD Eclipse Plus C8 2.1 × 50 mm, 1.8 µm (Agilent, Waldbronn, Germany) for separation. Further details can be found in the supplementary methods. Analysis and data representation were performed using MetaboAnalyst 6.0 [68] with probabilistic quotient normalised data.

### Flow cytometry

Mouse skin was dissected, washed once with 1x PBS, and then incubated at 37 °C for 1 h in 5 mg/ml Dispase II solution. The skin was washed with 1x PBS, and then the epidermis and dermis were separated. The separated epidermis and dermis were placed in GentleMACS MACS C tube containing 1 mg/ml Collagenase P and 20 μg/ml DNase I in DMEM/High glucose. Tissue was dissociated for 1 h using programme 37_Multi_B on the gentleMACS Octo Dissociator with heaters. Solutions were passed through 40 μm cell strainers and centrifuged at 400 × *g* for 5 min at 4 °C. Following washing, cells were blocked with FcR Blocking Reagent mouse (Miltenyi) with Zombie UV™ fixable Viability kit (BioLegend) in 0.5% BSA/PBS for 10 min on ice. Antibodies (supplementry information) were diluted in brilliant stain buffer (BD Biosciences, Franklin Lakes, New Jersey, USA) and incubated for 20 min on ice. Subsequently, cells were washed with 0.5% BSA/PBS, and then pellets were resuspended in FACS flow.

For cytokine analysis, mouse skin was ground under liquid nitrogen and resuspended in 2x PBS (w/v). Samples were rotated for 2 h at 4 °C. BD™ Cytometric Bead Array (CBA) Mouse Flex Sets (BD Biosciences) were used as per manufacturers’ recommendations.

Samples were measured using FACS Symphony A5SE (BD Biosciences). Analysis was performed using FlowJo v10.1 (BD Biosciences).

### In situ hybridisation

In situ hybridisation was performed with a mouse multiple normal organ tissue array (MOJ6221a-BX, Bio-Cat) with probe mm-Alox8 (1089301-C1, ACD-Bio) and BaseScope™ Assay Red kit (ACD-Bio) as per manufacturer’s recommendations.

### Immunofluorescence and histology staining

Mouse skin was dissected and washed with 1x PBS before formalin fixation for 24 h. Subsequently, tissue was dehydrated and embedded in paraffin, and 4 μm tissue sections were acquired. Following rehydration, heat-induced epitope retrieval was performed using AR6 or AR9 (Akoya Niosciences, Marlborough, Massachusetts, USA). Tissue sections were blocked with 1x antibody diluent/block (Akoya Biosciences) for 10 min, then incubated with primary antibodies for 30 min at room temperature. Following washing with 1x TBST, slides were incubated with Goat-anti Rabbit HRP secondary antibody (Agilent, Dako, Santa Clara, California, USA) at 1:500 for 10 min. HRP was detected using Opal fluorophores (Akoya Biosciences) for 10 min at room temperature. A subsequent heat-induced epitope retrieval step was performed to remove bound antibodies. Slides were then washed in PBS, and counterstaining with spectral DAPI (Akoya Biosciences) was performed. Multiplex staining for differentiation markers was performed as described above using Bond RX and Bond reagents (Leica). Further details can be found in supplementary information.

Masson goldner trichrome staining kit (3459, Carl Roth, Karlsruhe, Germany) was used as per manufacturer’s recommendations on 4 μm paraffin tissue sections.

TUNEL staining was performed with 7 μm tissue sections from snap-frozen tissue. Tissue sections were air-dried for 10 min and fixed with ethanol-glacial acetic acid (2:1) for 5 min. Click-iT™ Plus TUNEL Assay Kits for In Situ Apoptosis Detection (C10619; Invitrogen™) were utilised as per manufacturer’s instructions. Subsequently, slides were washed in PBS, and counterstaining with spectral DAPI (Akoya Biosciences) was performed.

### Lucifer yellow permeability assay

Lucifer Yellow was dissolved at 1 mg/ml in PBS. Mouse skin was dissected and washed once in PBS. Skin was incubated at 37 °C for 20 min with Lucifer Yellow solution placed onto the epidermis. Skin was washed with PBS before formalin fixation and paraffin embedding as described above. 10 µm tissue sections were rehydrated and incubated with DAPI.

### Microscopy

Slides were scanned with Vectra Polaris (Akoya Biosciences) equipped with PL-APO objectives at 20x (0.5 μm/pixel, 0.45 NA) or 40x (0.25 μm/pixel, 0.75 NA). Multispectral or brightfield images were acquired in qptiff format.

### Image analysis

Image analysis was performed in QuPath [69]. Manual annotations or a pixel classifier were used to segment the tissue. Mean pixel intensity for annotations was calculated using intensity features. Nuclei detection was performed with a script using StarDist plugin [70], and positive nuclei were calculated through threshold analysis. Subcellular spot detection was used to analyse BaseScope images. Image analysis scripts will be made available upon request.

### Statistics

All experiments performed had a minimum of three biological replicates from individual mice; the sample size was not pre-determined. Statistical analysis and data visualisation were performed using Prism (GraphPad, Boston, Massachusetts, USA). Data are presented as scatter plots with mean ± SEM, biological replicates represented by different symbol sets. Shapiro-Wilk testing was performed to determine data distribution and variance. Two-way ANOVAs were performed as stated in the figure legends. Statistical significance was described as *P ≤ 0.05, **P ≤ 0.01, ***P ≤ 0.001.

## Supplementary information

Supplementary Figures

Primer sequences

Antibodies

Orignal Western blots

Lipidomics methods

## Data Availability

Data is available upon request.
