# Supplementary material for: Alox8 knockout exacerbates imiquimod-induced psoriasis-like inflammation
Source: Cell Death Dis. 2026 Apr 10;17(1):390. doi: 10.1038/s41419-026-08727-9 (PMC13076715; doi:10.1038/s41419-026-08727-9)
Supplement: Supplementary file 1 — Supplementary Figures [file 41419_2026_8727_MOESM1_ESM.docx]

**Alox8 knockout exacerbates imiquimod-induced psoriasis-like inflammation**

Megan A. Palmer^1^, Rebecca Kirchhoff^2^, Lisa Hahnefeld^3,4,5^, Dominique Thomas^3,4,5^, Mohammed A. F. Elewa^1^, Xin You^1^, Blerina Aliraj^1^, Yvonne Benatzy^1^, Andreas Weigert^1,6^, Nils Helge Schebb^2^, Bernhard Brüne^1,7^

^1^Faculty of Medicine, Institute of Biochemistry I, Goethe University Frankfurt, Frankfurt, Germany

^2^Chair of Food Chemistry, School of Mathematics and Natural Sciences, University of Wuppertal, Wuppertal, Germany

^3^Goethe University Frankfurt, Faculty of Medicine, Institute of Clinical Pharmacology, Frankfurt, Germany

^4^Fraunhofer Institute for Translational Medicine and Pharmacology ITMP, Theodor-Stern-Kai 7, 60596 Frankfurt am Main, Germany

^5^Fraunhofer Cluster of Excellence for Immune Mediated Diseases CIMD, Theodor-Stern-Kai 7, 60596 Frankfurt am Main, Germany

^6^Department for Immunity of Inflammation, Mannheim Institute for Innate Immunoscience (MI3), Medical Faculty Mannheim, Heidelberg University, Mannheim, Germany.

^7^German Cancer Consortium (DKTK), Partner Site Frankfurt, Germany

Corresponding author: Megan A. Palmer, Goethe University Frankfurt, Faculty of Medicine, Institute of Biochemistry I, Theodor-Stern-Kai 7, 60590 Frankfurt, Germany. +49 69 - 6301-7424, [palmer@biochem.uni-frankfurt.de](mailto:palmer@biochem.uni-frankfurt.de)


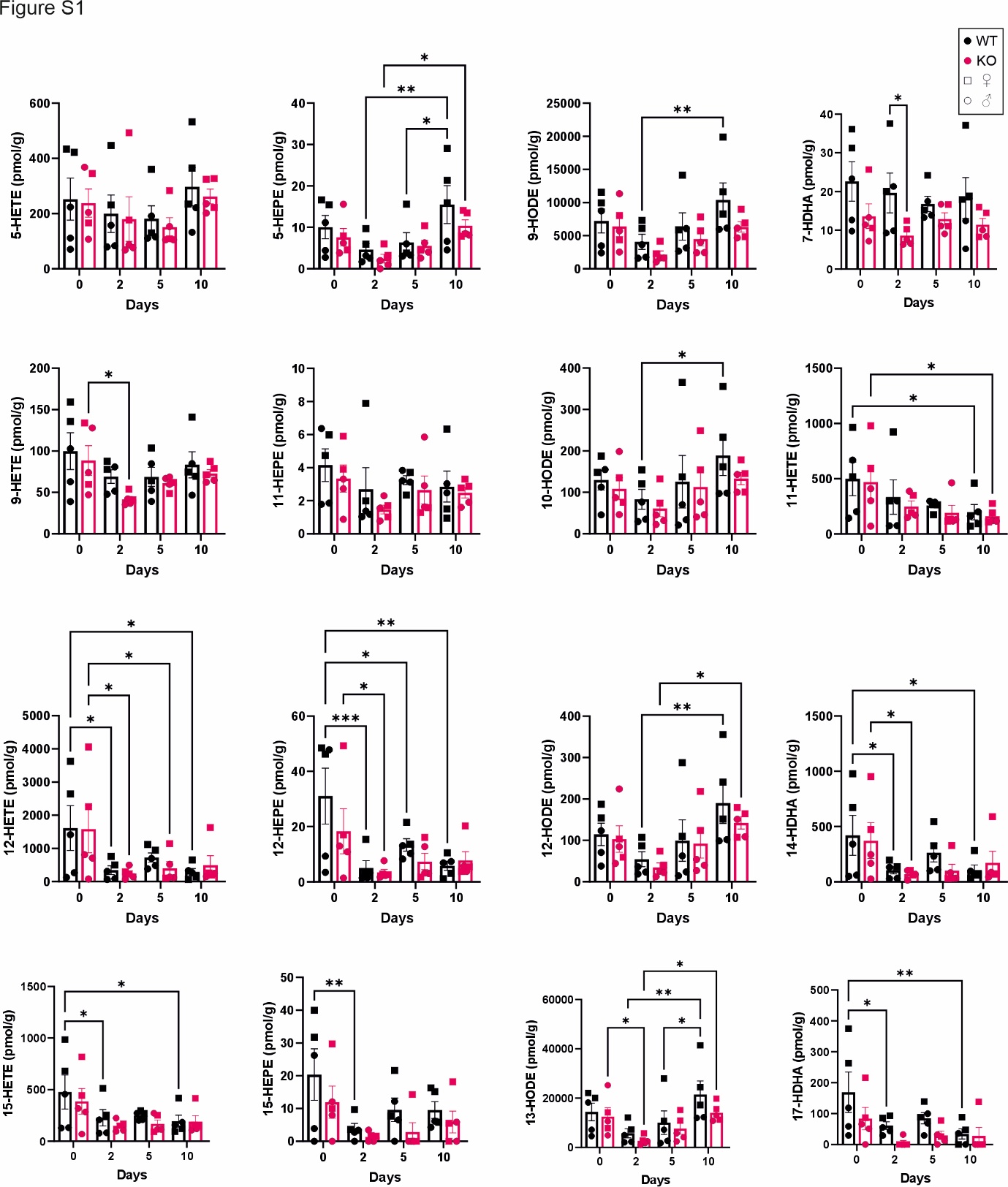


**Fig. S1. Oxylipins in Alox8 KO mouse skin.** IMQ was topically applied to the back skin of WT and Alox8 KO daily on day 0 for up to 6 times. Mice were sacrificed at day 0, 2, 5 or 10. Analysis of total oxylipins from mouse skin. Data are mean +/- SEM (N=5), two-way ANOVA was performed; significance denoted by * P ≤ 0.05, ** P < 0.01, *** P < 0.001.

**
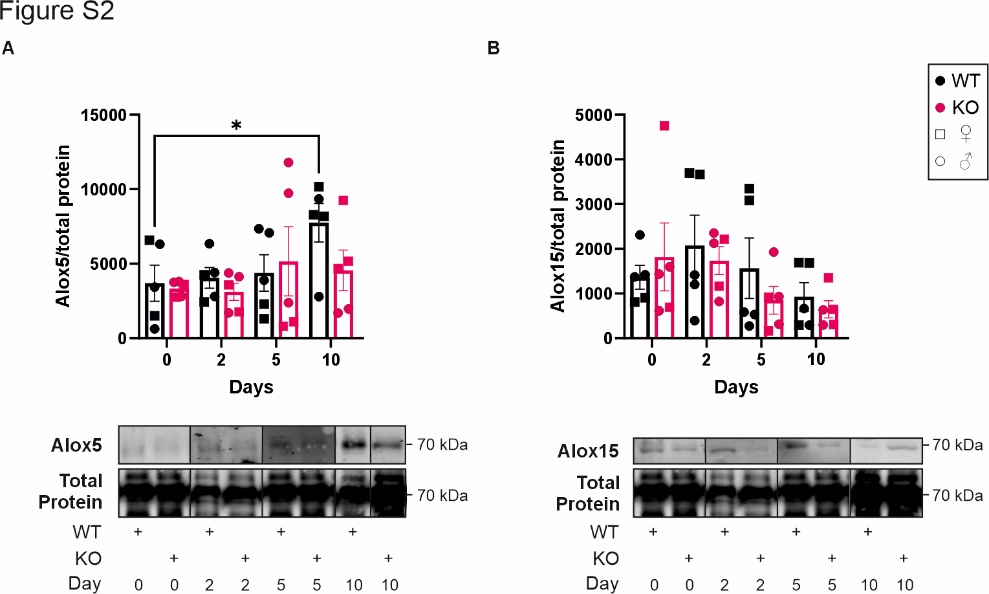
**

**Fig. S2. Protein expression of Alox5 and Alox15 in murine skin.** IMQ was topically applied to the back skin of WT and Alox8 KO daily on day 0 for up to 6 times. Mice were sacrificed at day 0, 2, 5 or 10. Western blot and densitometry analysis of (A) Alox5 and (B) Alox15 normalised to total protein. Data are mean +/- SEM, N=5, two-way ANOVA was performed; significance denoted by * P ≤ 0.05.

**
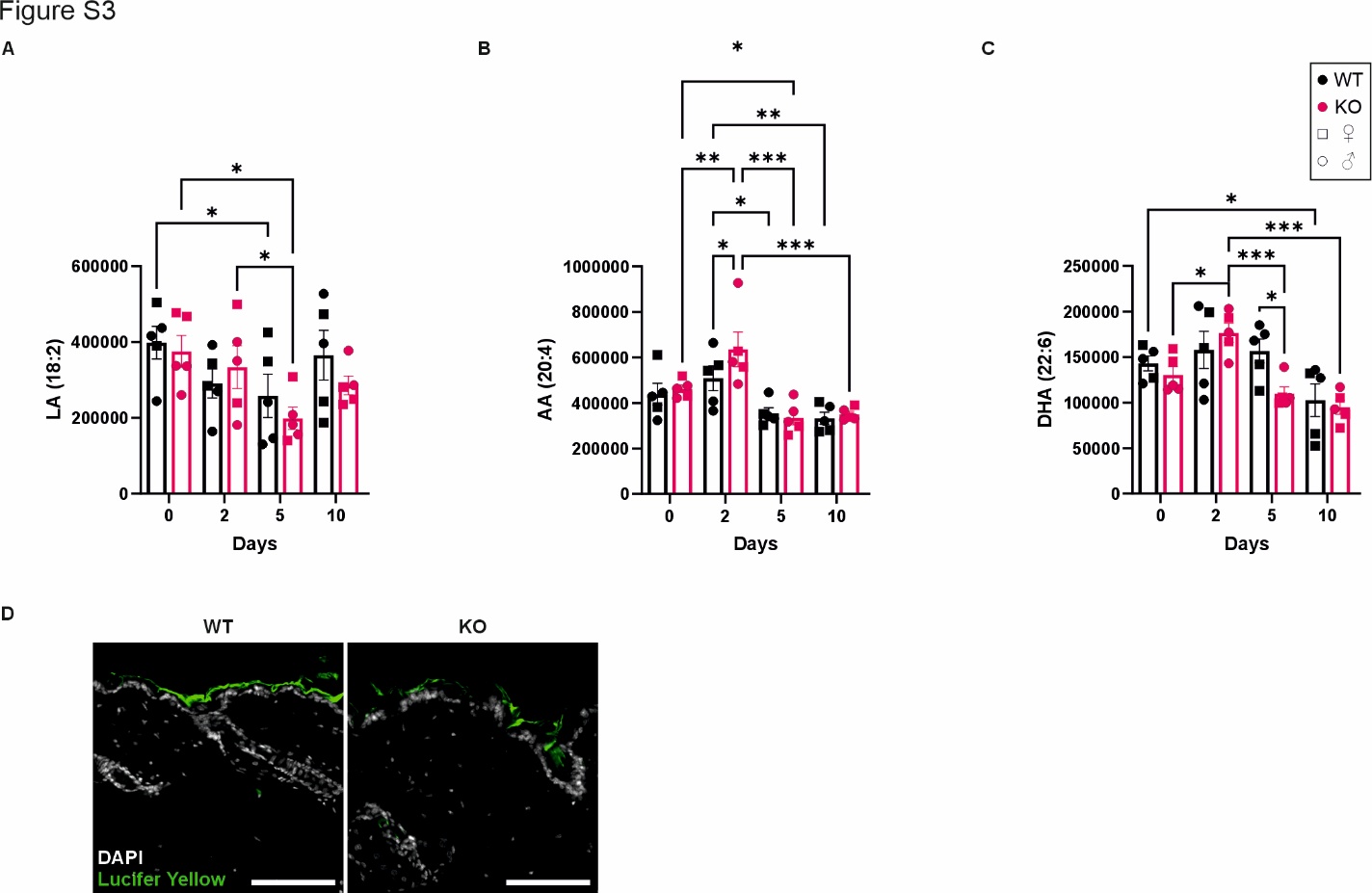
**

**Fig. S3. Lipid abundance of PUFAs in Alox8 mouse skin.** IMQ was topically applied to the back skin of WT and Alox8 KO daily on day 0 for up to 6 times. Mice were sacrificed at day 0, 2, 5 or 10. Abundance of polyunsaturated fatty acids (A) linoleic acid (LA), (B) arachidonic acid (AA) and (C) docosahexaenoic acid (DHA). Data are mean +/- SEM of area under the curve, N=5, two-way ANOVA was performed; significance denoted by * P ≤ 0.05, ** P < 0.01, *** P < 0.001.

**
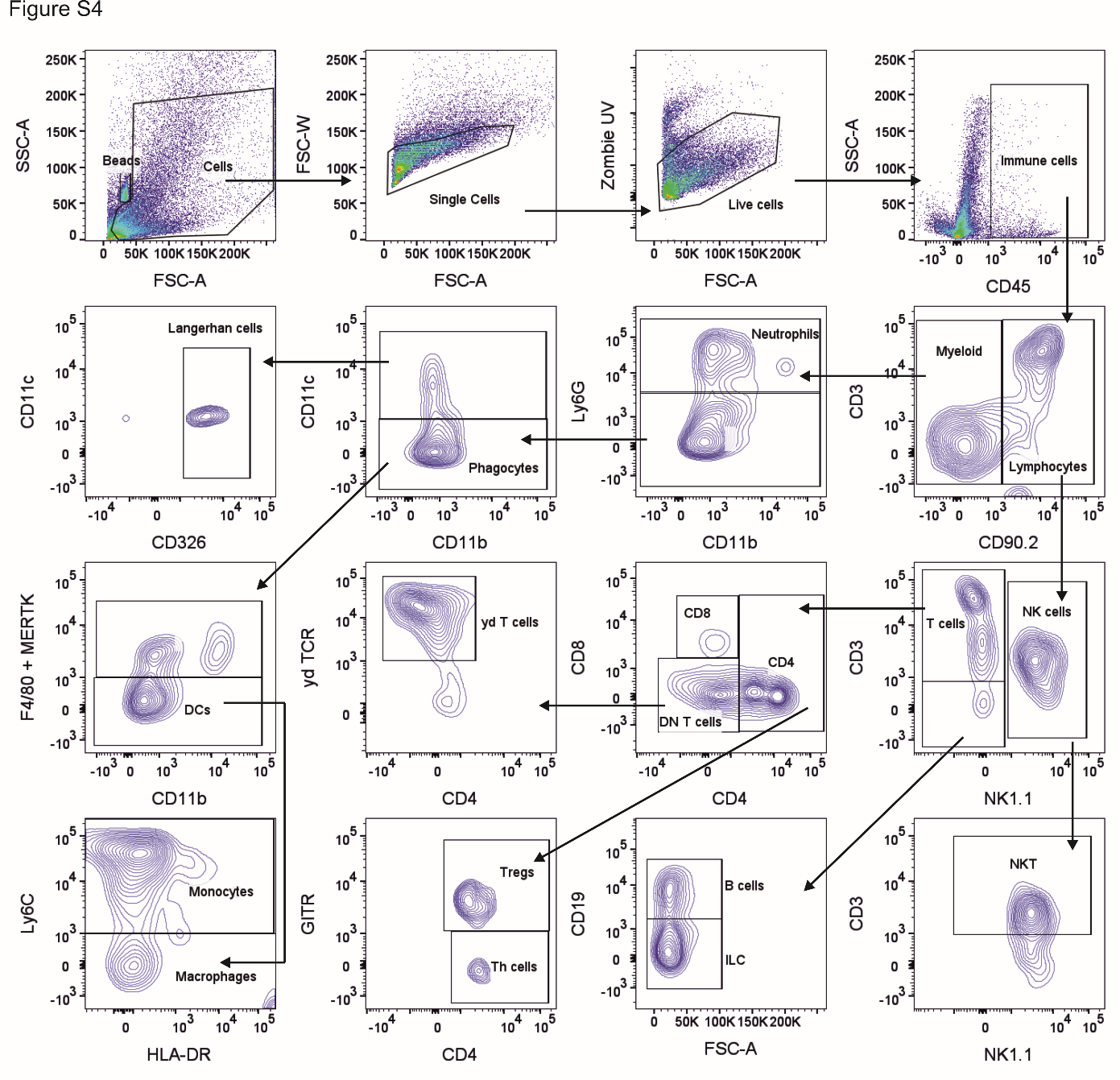
**

**Fig. S4. Gating strategy for flow cytometry.** Gating strategy for flow cytometry in Fig.6, Fig.7 and Fig. S5.


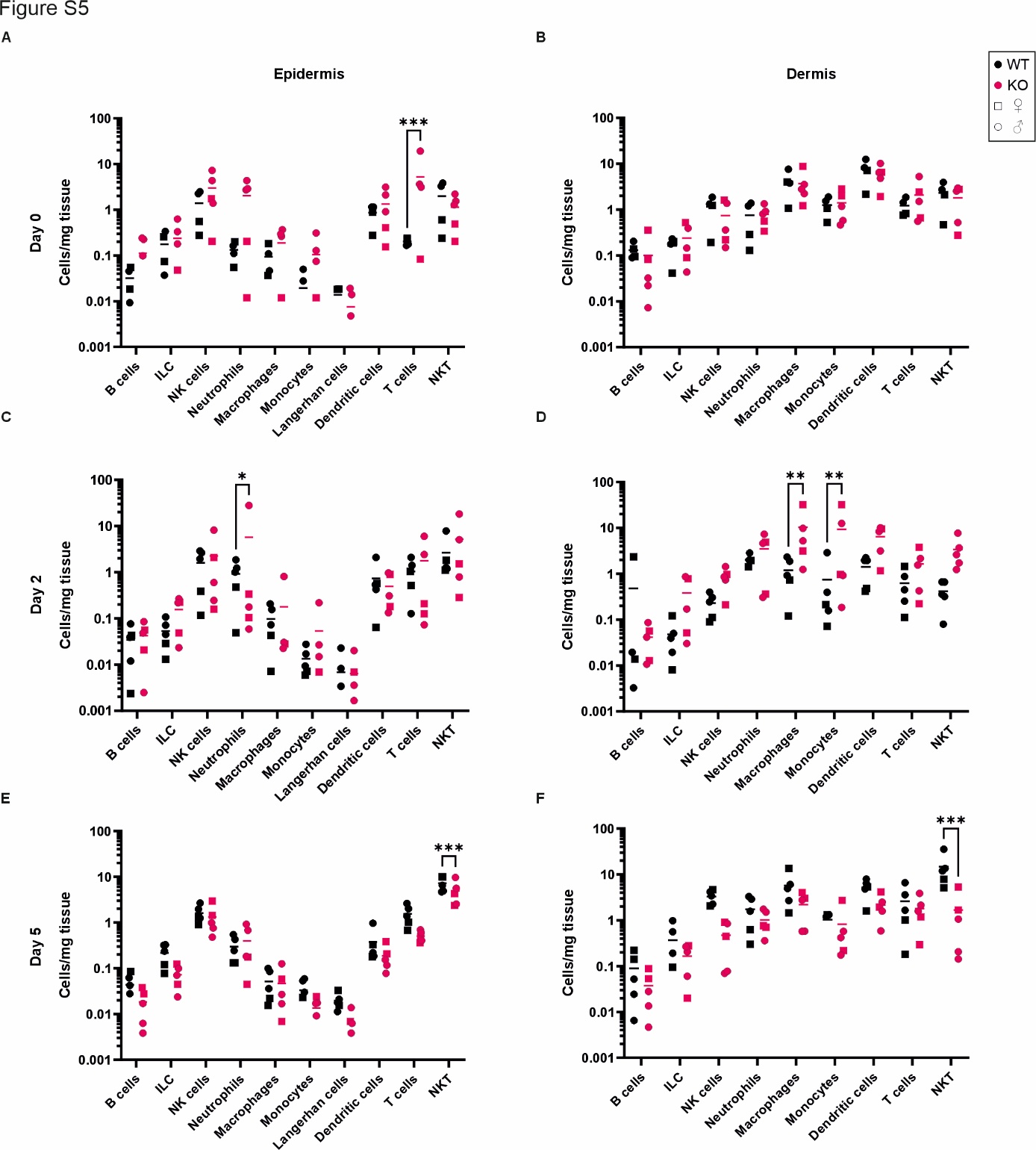


**Fig. S5. Immune cell profile in epidermis and dermis of murine skin.** IMQ was topically applied to the back skin of WT and Alox8 KO daily on day 0 for up to 5 times. Mice were sacrificed at day 0, 2, or 5. Flow cytometry analysis of single cell suspensions of epidermis (A,C,E) or dermis (B,D,F). Data are mean number of cells per mg of tissue, N=5 (except for WT day 1), two-way ANOVA was performed; significance denoted by * P ≤ 0.05, ** P < 0.01, *** P < 0.001.

**
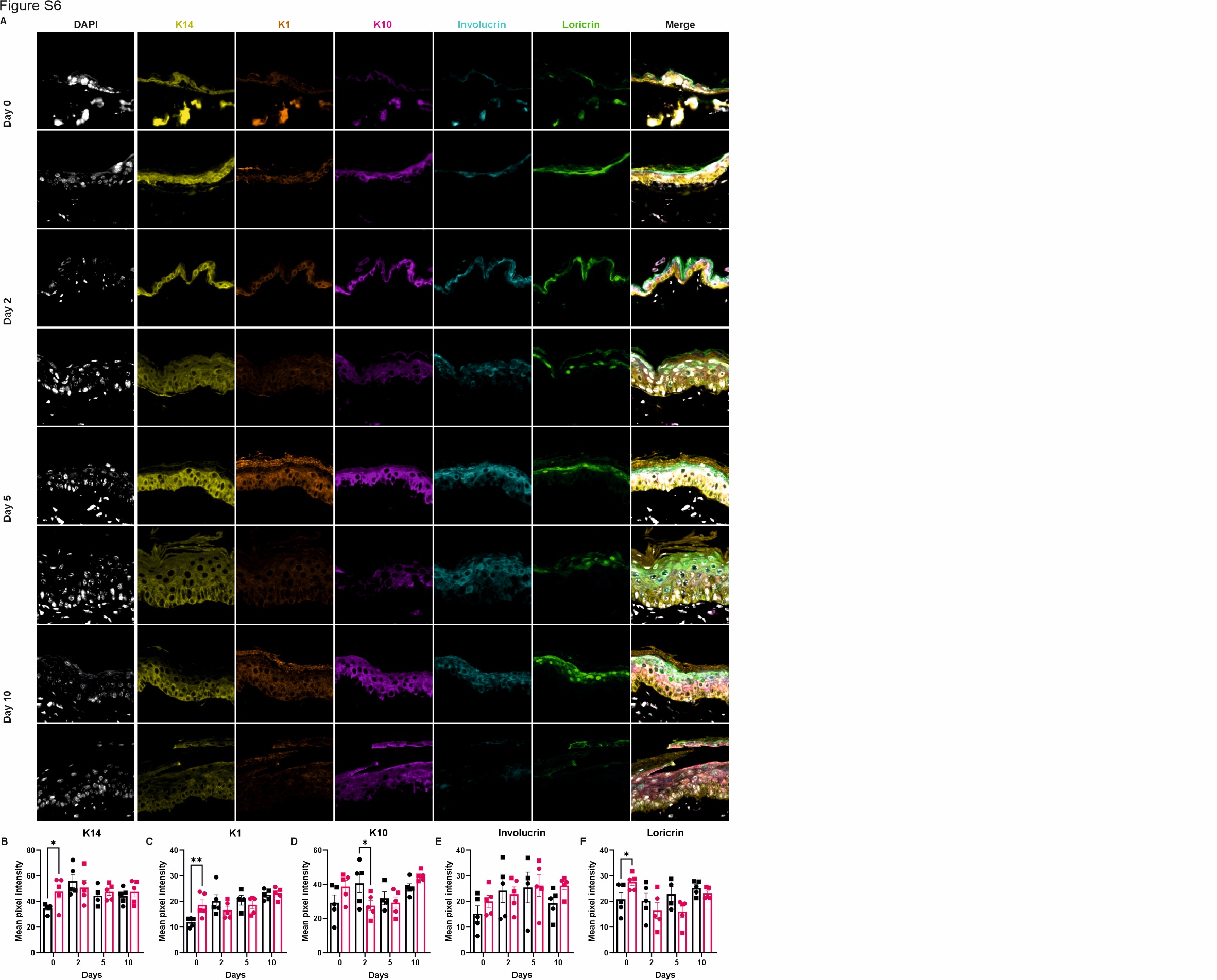
**

**Fig. S6. Individual channel images for differentiation markers.** IMQ was topically applied to the back skin of WT and Alox8 KO on day 0 for up to 6 days. Mice were sacrificed at day 0, 2, 5 or 10. Individual immunofluorescence images of differentiation markers from Fig. 8A, scale bar 20 μm. Image analysis of mean pixel intensity of (B) K14, (C) K1, (D) K10, (E) Involucrin and (F) Loricrin. Data are mean +/- SEM, N=5, two-way ANOVA was performed; significance denoted by * P ≤ 0.05.


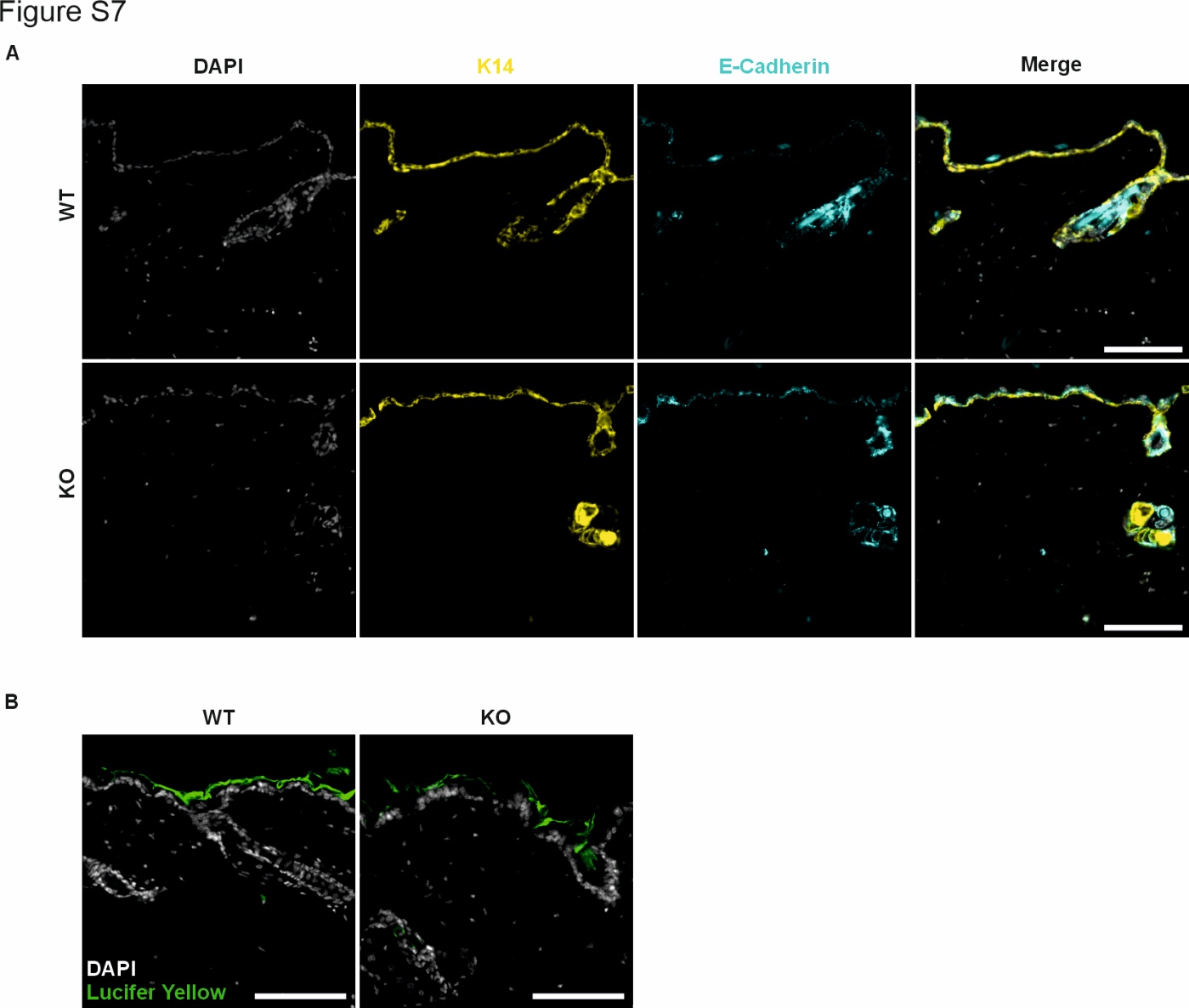


**Fig. S7. Epidermal barrier is not disrupted in Alox8 mice.** WT and Alox8 KO mice skin stained with (A) K14 and E-cadherin immunofluorescence and (B) Lucifer yellow.


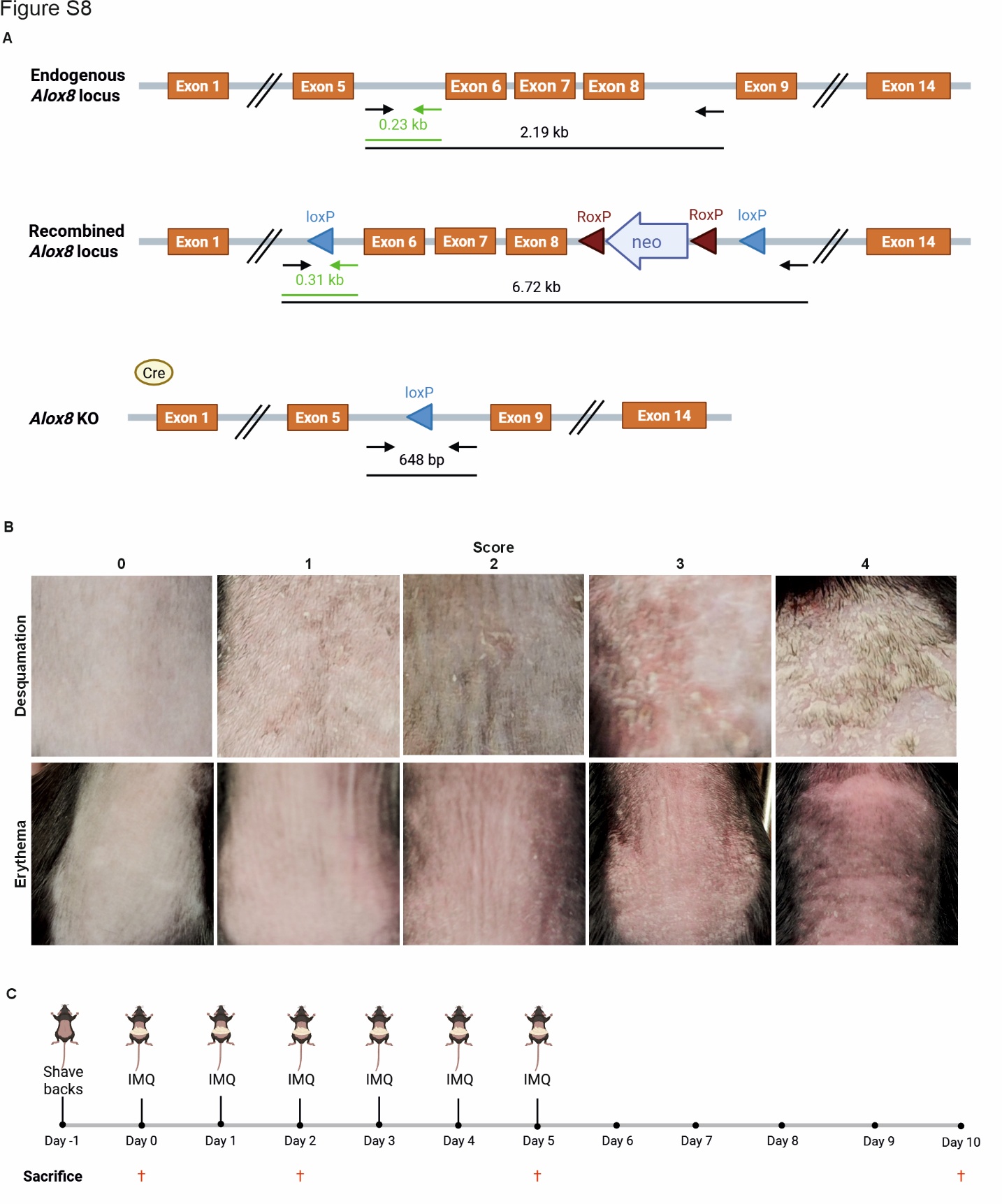


**Fig. S8. Murine experimental methods.** (A) Alox8 KO recombined locus and genotyping strategy. Primers used for genotyping are indicated with black and green arrows, corresponding product lengths are indicated. (B) Example images of desquamation and erythema for PASI scoring. (C) IMQ application timeline. Created in BioRender. Palmer, M. A. (2026) https://BioRender.com/tza77ls
