# Supplementary material for: Alox8 knockout exacerbates imiquimod-induced psoriasis-like inflammation
Source: Cell Death Dis. 2026 Apr 10;17(1):390. doi: 10.1038/s41419-026-08727-9 (PMC13076715; doi:10.1038/s41419-026-08727-9)
Supplement: Supplementary file 4 — Orignal Western blots [file 41419_2026_8727_MOESM4_ESM.pdf]

Fig.S1B

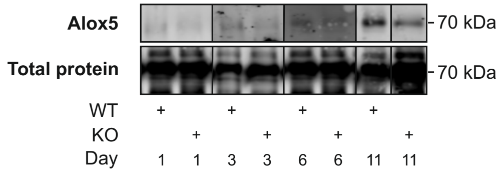

Total protein

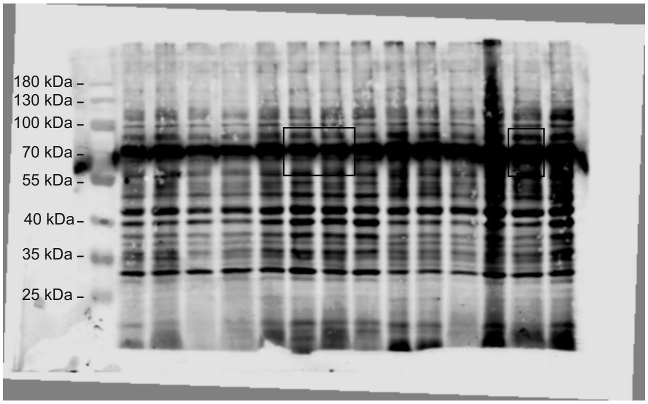

Alox5

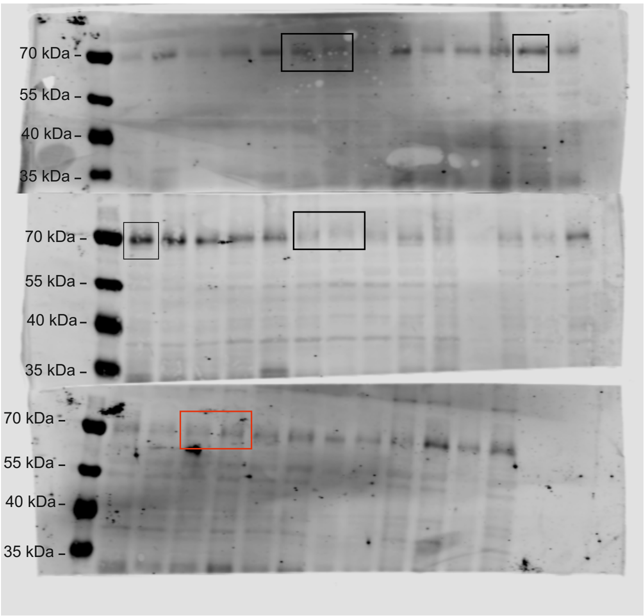

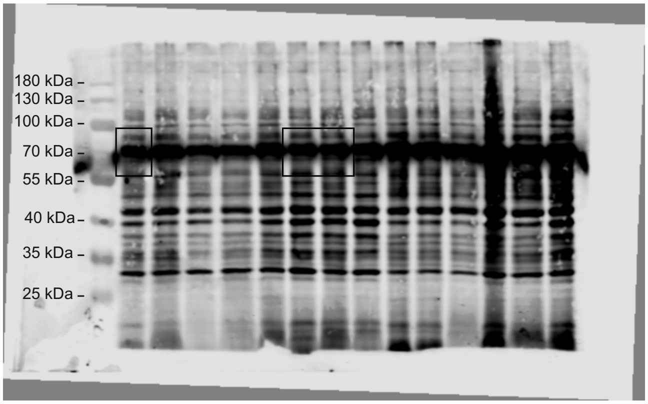

* Red box represents flipped image

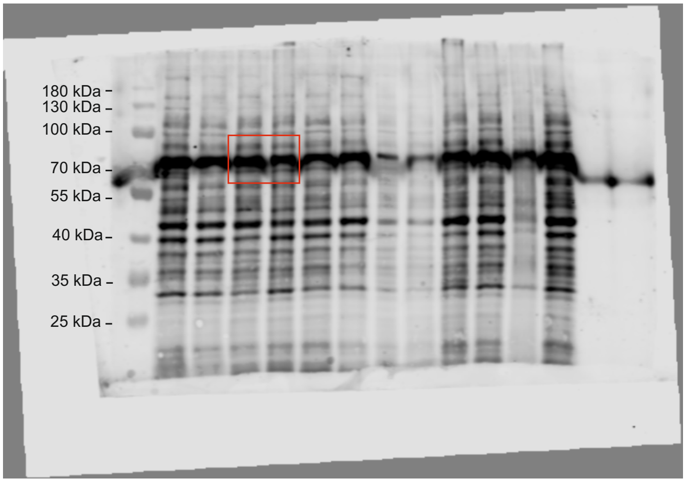

Fig.S1B

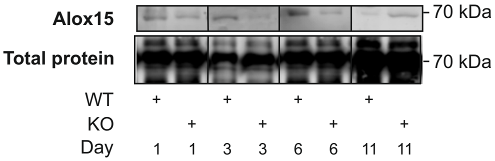

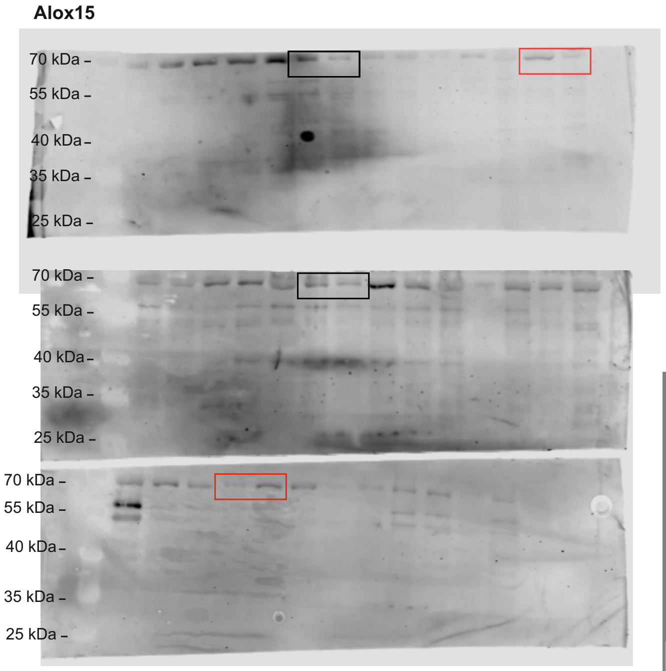

* Red box represents flipped image

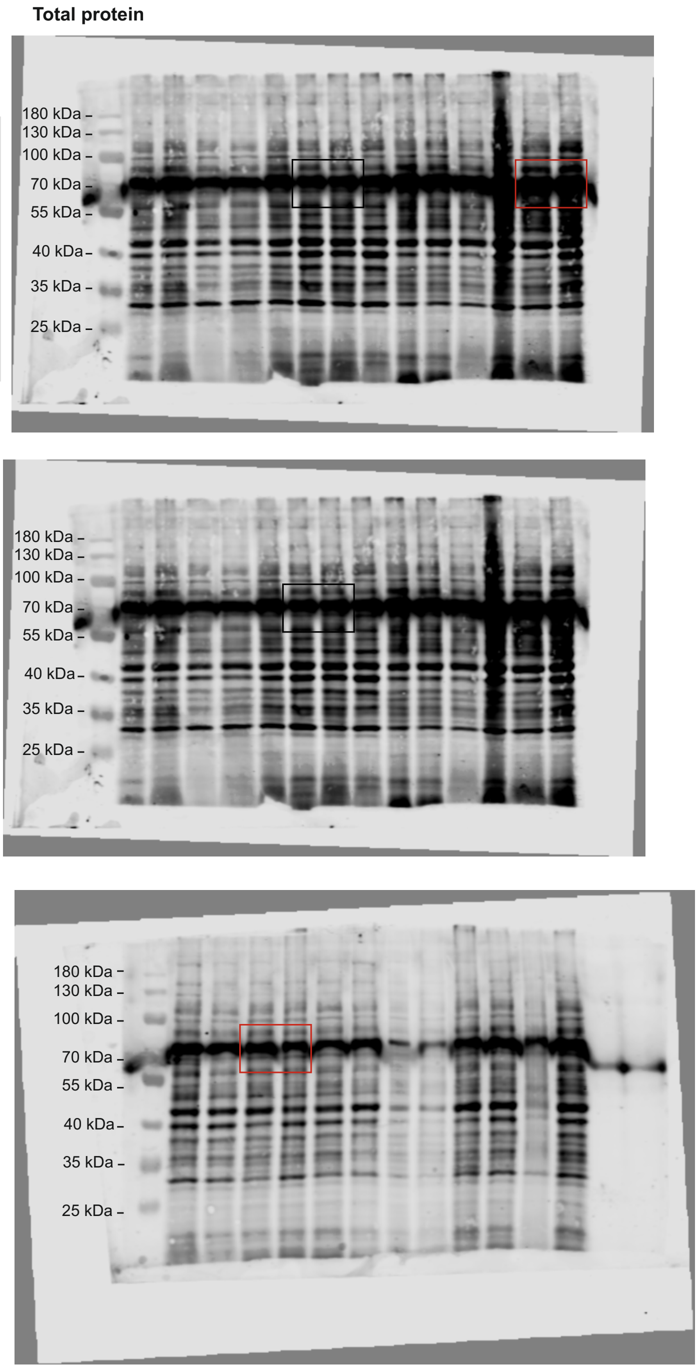

Fig.5

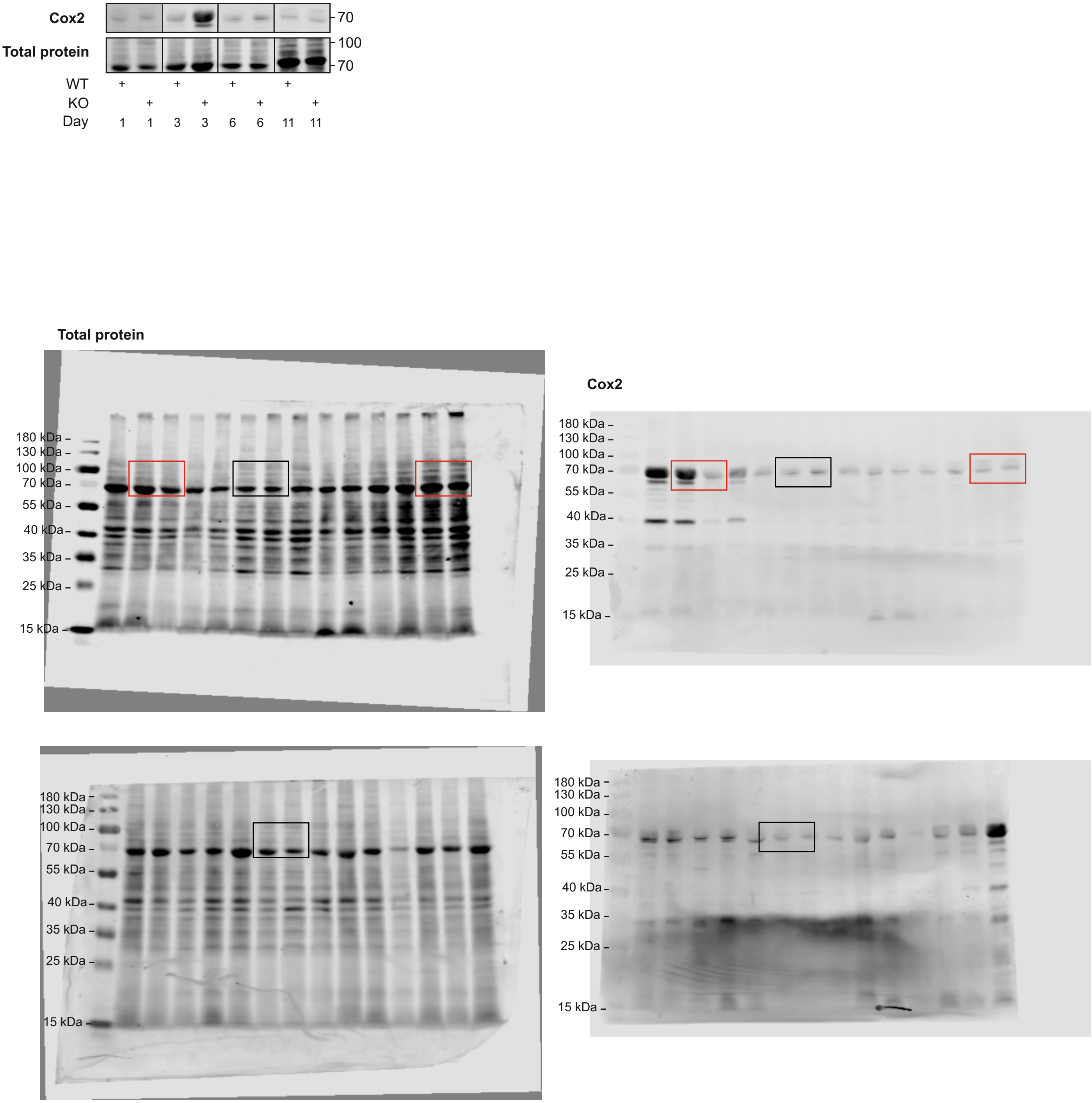

* Red box represents flipped image
